# On-tissue derivatization for mass spectrometry imaging reveals the distribution of short chain fatty acids in murine digestive tract

**DOI:** 10.3389/fcimb.2025.1584487

**Published:** 2025-10-03

**Authors:** Kaoru Nakagawa, Mami Okamoto, Masako Nishida, Kenta Terashima, Manami Kobayashi, Kanae Teramoto, Akiko Kubo

**Affiliations:** ^1^ Division of Analutical & Measuring Instruments, Shimadzu Corporation, Kyoto, Kyoto, Japan; ^2^ Division of Dermatology, Department of Internal Medicine, Graduate School of Medicine, Kobe University, Kobe, Hyōgo, Japan

**Keywords:** short-chain fatty acids, SCFAs, MALDI, MSI, TMPA, HATU, proteotyping

## Abstract

Short-chain fatty acids (SCFAs), which are produced by microorganisms in the digestive tract of animals, play an important role in maintaining homeostasis in the host, including immune function. Different types of SCFAs are produced by different intestinal bacterial communities. However, visualizing their spatial distribution within tissue sections has been difficult. This is primarily due to the volatility of SCFAs, which makes detection challenging, even with matrix-assisted laser desorption/ionization (MALDI) mass spectrometry imaging (MSI) using an atmospheric pressure ion source. To address this issue, we minimized the volatility of SCFAs in fresh tissue sections. Then, we used N,N,N-trimethyl-2-(piperazin-1-yl)ethan-1-amine iodide (TMPA) and 1-((dimethylamino)(dimethylimino)methyl)-1H- [1,2,3]triazolo[4,5-b]pyridine-3-oxide hexafluorophosphate (HATU) to chemically derivatize the carboxylic acid into a quaternary amine. This *in situ* derivatization enabled visualization of SCFAs using MALDI-MSI. In the cecum of mice, strong signals for butyrate and propionate were detected in areas with high bacterial density, as identified by hematoxylin staining. This indicates that these SCFAs are produced by bacteria. Anaerobic bacteria were cultured from the cecum of another individual raised under the same environment. Strain identification was performed using MALDI mass spectrometry of bacterial protein finger prints which confirmed the presence of bacteria that produce SCFAs. This approach, which combines minimizing volatility and *in situ* derivatization, provides a powerful tool for elucidating the spatial relationship between intestinal bacteria and metabolites including SCFAs.

## Introduction

Short-chain fatty acids (SCFAs) are volatile, low molecular weight compounds primarily produced by microorganisms residing in the gut. While dietary monosaccharides are digested and absorbed by host digestive enzymes (Markowiak-Kopec and Slizewska, 2020), indigestible dietary fibers and oligosaccharides are degraded and metabolized by the intestinal flora. This microbial metabolism results in the production and accumulation of SCFAs, such as acetic acid, propionic acid and butyric acid, in the gastrointestinal tract (Dalile et al., 2019; Myhrstad et al., 2020). In recent years, numerous studies have reported a variety of effects of SCFAs in host physiology, highlighting their particularly important contributions to immune system regulation (Silva et al., 2020; Mann et al., 2024).

SCFAs are volatile substances and cannot be detected by mass spectrometers equipped with vacuum ion sources (Harada et al., 2009). Even with devices equipped with atmospheric pressure ion sources, detection of carboxylic acids remains difficult. This is because no appropriate matrix for efficiently ionizing low-molecular-weight carboxylic acids is known (Krivosheina et al., 2021). Various on-tissue derivatization methods have been investigated to visualize metabolites containing carboxyl groups in fresh frozen sections by MALDI-mass spectrometry imaging (MSI) (Sun et al., 2020; Krivosheina et al., 2021; Zhou et al., 2021; Cohen et al., 2023; Forsman et al., 2023; Han et al., 2023; Kaya et al., 2023; Bao et al., 2024). Sun et al. reported that TMPA covalently binds to carboxylic acids on tissue sections in the presence of HATU and 1-hydroxybenzotriazole (HOBt) and can be detected by MALDI-MSI. Zemaitis et al. demonstrated that using 1-ethyl-3-(3-dimethylaminopropyl)-carbodiimide (EDC), an activator of carboxylic acids, and 4-(2-((4-bromophenethyl)dimethylammonium)ethoxy)benzenaminium dibromide (4-APEBA) enabled visualization of organic acid distribution in plant tissues by MALDI-MSI (Zemaitis et al., 2023). Han et al. successfully visualized volatile carboxylic acids using MALDI-MSI by fixing mouse digestive tract tissue to slides precoated with reagents necessary for derivatization and exposing it to derivatization reagents prior to drying (Han et al., 2023). Their method is an excellent approach that prevents volatilization while enhancing the detection efficiency of short chain fatty acids. This is achieved by bringing volatile carboxylic acids contained in frozen tissue into contact with derivatization reagents before they diffuse into the ambient air at room temperature. Their method is effective when a cryostat for creating fresh frozen sections and conductive slides pre-coated with derivatization reagents can be prepared in the same laboratory. However, in many cases, frozen sections on conductive slides are stored frozen and sent to the laboratory for MALDI-MSI analysis. Therefore, we modified the method of Sun et al (Sun et al., 2020). to develop a reproducible method for detecting SCFAs in animal tissues using chemical derivatization on tissue and MALDI-MSI. This approach successfully analyzed the distribution of low molecular weight carboxylic acids in the contents of the mouse small intestine and cecum.

## Materials

Diethyl ether, acetonitrile (AcCN), acetone, 10%-formaldehyde neutral buffer solution, and formic acid were purchased from Nacalai tesque (Kyoto, Japan). 1-(2-Dimethylaminoethyl)piperazine, iodomethane, and O-(7-azabenzotriazol-1-yl)-N,N,N’,N’-tetramethyluronium hexafluorophosphate (HATU), ultra-Pure water and carboxymethylcellulose were purchased from FUJIFILM Wako Chemical (Osaka, Japan). 4-methylmorpholine (4-MM), 1,5-naphthalenediamine (1,5-DAN) and standard samples of carboxylic acids were purchased from Tokyo Chemical Industry (Tokyo, Japan). Carrazzi’s hematoxylin and pure eosin were purchased from Muto Pure Chemicals (Tokyo, Japan). Alpha-Cyano-4-hydroxycinnamic acid (CHCA) was purchased from Sigma Aldrich (St. Louis, MO, USA). Eight weeks old C57BL/6NJ mice were purchased from The Jackson Laboratory (Kanagawa, Japan). ITO-coated glass slides (SI0100M) were purchased from Matsunami Glass (Osaka, Japan). Brucella HK agar plates were purchased from Kyokuto pharmaceutical (Tokyo Japan). AnaeroPouch®-Anaero were purchased from Mitsubishi Gas Chemical (Tokyo, Japan).

## Methods

### Synthesis of TMPA

The synthesis of TMPA was performed as described by Sun et al (Sun et al., 2020), and the structure of the synthesized product was confirmed by high-resolution MS and MS/MS spectra. A total of 2.36 g (15 mmol) of 1-(2-dimethylaminoethyl) piperazine and 0.71 g (5 mmol) of iodomethane were each dissolved in 100 mL of diethyl ether. The solutions were then mixed at room temperature. After stirring for 1 hour, the resulting white precipitate was collected by filtration, rinsed five times with diethyl ether, and dried. The dried powder was ground in a mortar and used in subsequent experiments.

### Frozen tissue section preparation

All animal experiments were approved by the Ethics Committee of Kobe University (P211104). The small intestine and cecum of mice were collected after sacrifice under deep anesthesia. Fresh tissues were frozen in 2% carboxymethylcellulose solution. Serial tissue section, 10-μm thick, were prepared using a cryostat microtome (Tissue-tek Polar B, Sakura-Finetek, Tokyo, Japan) at -20°C and mounted on ITO-coated glass slides. The tissue sections were kept in the cryostat chamber for 2 days to dry. The glass slides were stored at -80°C in 50 mL conical tubes containing silica gel. Prior to use, the slides were brought to room temperature inside the closed tubes, then used for on-tissue chemical derivatization. Condensation must be prevented from forming on the slide glass to which the tissue section is attached. The tissue section must never be dried in a vacuum.

### On-tissue chemical derivatization

A 10 mL solution containing TMPA (2 mM), HATU (2 mM), and 4-MM (2 mM) in AcCN was applied to the tissue sections using iMLayer™ AERO (Shimadzu Corp. Kyoto, Japan) (Lu et al., 2023). The nozzle-to-target distance, track speed, and track spacing were set to 5 cm, 40 mm/sec, and 1 mm, respectively. The drying time between sprays was 1 sec. Nozzle nitrogen gas pressure was set at 0.2 MPa. A resistance tube label c (Shimadzu, Kyoto, Japan) was used. Slides were incubated in a closed container saturated with AcCN gas at 23 °C for 4 hours.

### Matrix application

For matrix deposition, 20 mg/mL of 1,5-DAN in methanol was sprayed using an airbrush (Proconboy FWA Platinum 02 double action: PS270, GSI Creos. Tokyo, Japan), or CHCA (300 mg) was sublimated at 250 °C under vacuum (5 × 10^−2^ Pa) using the iMLayer™ (Shimadzu, Kyoto, Japan), a matrix deposition device that can control the temperature and calculate the matrix layer thickness during sublimation. The iMLayer was used to monitor and ensure a uniform layer of 0.7 μm thickness (Morikawa-Ichinose et al., 2019).

### MALDI-MSI

MSI experiments were performed using the iMscope™ QT with LCMS™-9030 (Shimadzu Corp. Kyoto, Japan) (Nakagawa et al., 2024). Mass calibration was performed using DHB cluster ions and angiotensin II before beginning the analysis of the specimen. The instrument features precise temperature control to ensure mass accuracy during analyses. The spatial resolution was set to 10, 25, and 50 μm by adjusting the laser pitch and diameter at the focus point. The laser was fired 50 times per data point at 1000 Hz. MALDI mass spectra were acquired in positive ion mode over an *m/z* range of 200-500. The desolvation line (DL) temperature was set to 290°C. MS images were superimposed with and HE stained images and visualized using IMAGEREVEAL™ MS software (Kubo et al., 2023).

### On-tissue MALDI-MS/MS experiment

The on-tissue MS/MS experiment was performed on an iMscope™ QT with LCMS™-9030 instrument (Shimadzu Corp. Kyoto, Japan) (Nakagawa et al., 2024) to confirm the structure of TMPA-derivatized fatty acids. The laser repetition rate was set to 1000 Hz and each MS/MS spectrum was the sum of 5000 laser shots for precursor and product ions. MS/MS spectra were acquired with Q1 resolution of 5.0 u, collision energy of 30.0, and CE spread of +/-10. The spectra obtained by MS/MS analysis were verified by comparison with the reference (Sun et al., 2020; Han et al., 2023).

### LC-QTOF MS experiment

Flow injection analysis was performed using a Nexera™ X3 system coupled with a LCMS™-9030 (Shimadzu Corp., Kyoto, Japan). The LC conditions were as follows: The mobile phase (A) consisted of 0.1% formic acid in water, and the mobile phase (B) consisted of acetonitrile. The injection volume was 0.1 µL, the flow rate was 0.3 mL/min, and the run was performed with 50% mobile phase B. The MS conditions were as follows: nebulizing gas flow: 2.0 L/min; heating gas flow: 10.0 L/min; drying gas flow: 10.0 L/min; interface temperature: 300 °C; DL temperature: 250 °C; heat block temperature: 400 °C; CID gas: 230 kPa; TOF range: *m/z* 50–1000; event time: 100 ms for MS scan and 100 ms for MS/MS; and interface voltage: 4 kV.

### Hematoxylin and eosin staining

After raster scanning for MSI, the slides were washed with acetone for 15 minutes, followed by fixation in a 10% formaldehyde neutral buffer solution for an additional 15 minutes. Hematoxylin and eosin staining was performed according to the manufacturer’s protocol (Muto Pure Chemicals, Tokyo, Japan). The HE-stained slides were scanned using a Nanozoomer virtual slide scanner (Hamamatsu Photonics, Hamamatsu, Japan).

### MSI DATA analysis

All spectral data were processed using IMAGEREVEAL™ MS software (Shimadzu Corp., Kyoto, Japan). Light microscopy images of HE-stained tissue sections were superimposed on the MS^1^ images, and spectral data from selected regions were exported for further analysis. Marvin was used for drawing, displaying and characterizing chemical structures, substructures and reactions, Marvin 17.21.0, Chemaxon (https://www.chemaxon.com).

### Identification of intestinal bacteria

The contents of the cecum of C57BL/6N mice raised in the same environment as the mice used in the MALDI-MSI analysis were collected. The cecal contents were suspended in an equal amount of PBS, and this solution was diluted 10-fold with PBS in a stepwise manner up to 10^8 times (Slack and Wheldon, 1978). Ten microliters of this solution were spotted on Brucella HK medium. The plates were sealed in a container with an AnaeroPouch-Anaero® and incubated anaerobically at 35 °C for 48 hours, after which colonies were observed. Single colonies were grown on a new Brucella HK medium and used for identification. The isolated single colonies were picked up with a clean toothpick and applied to the analysis plate. After drying, 0.5μL of 80% formic acid was spotted and air-dried. A 1 μL spot of CHCA matrix solution was applied and dried further. For bacterial protein analysis, MALDI-TOF MS measurements were performed in positive linear mode using MALDI-8030 EasyCare (Shimadzu Corporation, Japan) equipped with a 200 Hz Nd: YAG laser (355 nm) and 60 Hz nitrogen laser(337nm). Before sample analysis, the MALDI-TOF MS instrument was mass-calibrated externally using six peaks with m/z 4,365.4, 5,381.4, 6,411.6, 7,274.5, 8,369.8, 10,300.1, 11,450.3, 13,651.3, and 16,019.6 from *Escherichia coli* DH5α. Three individual mass spectra were acquired for each bacterial extract in the range of m/z 4,000–20,000. The results were compared with the database using the MicrobialTrack software (Shimadzu corp. Kyoto, Japan).

## Results

**Figure 1:**
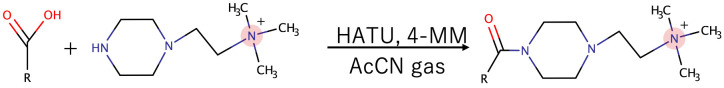
Synthesis reaction of TMPA-conjugated carboxylic acid.

The metabolic reactions mediated by microorganisms in the gastrointestinal tract are highly complex, and the metabolites produced by these microorganisms play an important role in maintaining host homeostasis, immune regulation, and defense mechanisms. In this study, a method was developed to simultaneously detect carboxyl-containing metabolites, including volatile organic acids, to compare the localization of microorganisms in the gut and the distribution of metabolites, such as SCFAs, on the same tissue section. First, a condensation reaction was performed in the liquid phase between several carboxyl-containing compounds present *in vivo* and the quaternary amine TMPA. The resulting products were confirmed by LC-MS and MALDI-MS. HATU was used as a coupling reagent to activate the carboxyl groups into active esters, allowing TMPA to condense with various carboxylic acid compounds in the presence of the organic amine 4-MM under AcCN gas (**Figure 1**). The target monocarboxylic acids, their theoretical exact masses after derivatization, and the detected *m/z* values are summarized in **Table 1**. Monocarboxylic acids reacted well with TMPA and were sensitively detected by LC-MS and MALDI-MS. In contrast, polycarboxylic acids such as citric acid did not undergo complete derivatization under the current reaction conditions. Molecules with one or two unreacted carboxyl groups were observed to coexist (**Supplementary Figure 1**, **Table 2**). Furthermore, although LC-MS analysis confirmed the presence of molecules with two carboxyl groups derivatized with the quaternary amine, MALDI-MS analysis detected only molecules with a single derivatized carboxyl group. Molecules with more than two derivatized carboxyl groups were not detected in the MALDI-MS analysis.

**Table 1:** Types of TMPA-derivatized monocarboxylic acids, their theoretical values, and the actual MS^1^ values measured from mouse intestines.

Target compound	Theoretical value	Measurement value (MS^1^)
Propionic acid	228.2071	228.2071
Lactic acid	244.2020	244.2019
Butyric acid	242.2227	242.2229
Pentanoic acid	256.2384	256.2376
Hexanoic acid	270.2540	270.2534
Octanoic acid	298.2853	298.2850
Decanoic acid	326.3166	326.3170
Lauric acid	354.3479	354.3446
Myristic acid	382.3792	382.3788
Palmitoleic acid	408.3949	408.3949
Palmitic acid	410.4105	410.4108
Linolenic acid	432.3949	432.3943
Linoleic acid	434.4105	434.4108
Oleic acid	436.4262	436.4262
Stearic acid	438.4418	438.4399
Arachidic acid	466.4731	466.4732
Arachidonic acid	458.4105	458.4115
Eicosapentaenoic acid	456.3949	456.3952
Docosahexaenoic acid	482.4105	482.4108

**Table 2:** Theoretical and measured *m/z* values of TMPA-derivatized citric acid.

Target compound	Theoretical value	Measurement value
TMPA-derivatized citric acid (monovalent)	287.1232	287.1227
TMPA-derivatized citric acid (divalent)	191.1122	191.1094
TMPA-derivatized citric acid (trivalent)	159.1063	159.1038

This table shows precursors ions corresponding to citric acid derivatized with TMPA at one, two, or three carboxyl groups.

### MALDI-MSI experiments of tissue sections subjected to on-tissue chemical derivatization

The organic amine 4-MM and HATU were used as condensing agents for derivatization on tissue sections, followed by MALDI-MSI detection. First, OTCD was performed according to the previously reported method (Sun et al., 2020), followed by MALDI-MSI analysis. No peaks originating from volatile short-chain fatty acids were detected at that time. Since the slides were evaporated prior to derivatization, it was hypothesized that volatile carboxylic acids had been lost from the tissue. Therefore, we developed a method to remove frozen sections containing volatile substances from the cryostat while preventing surface condensation. Although tissue sections and embedding materials contain moisture and volatile substances, leaving them in the cryostat allows some moisture to sublimate. Upon transfer to room temperature, this moisture can be adsorbed by silica gel placed in a 50 mL conical tube.

**Figure 2:**
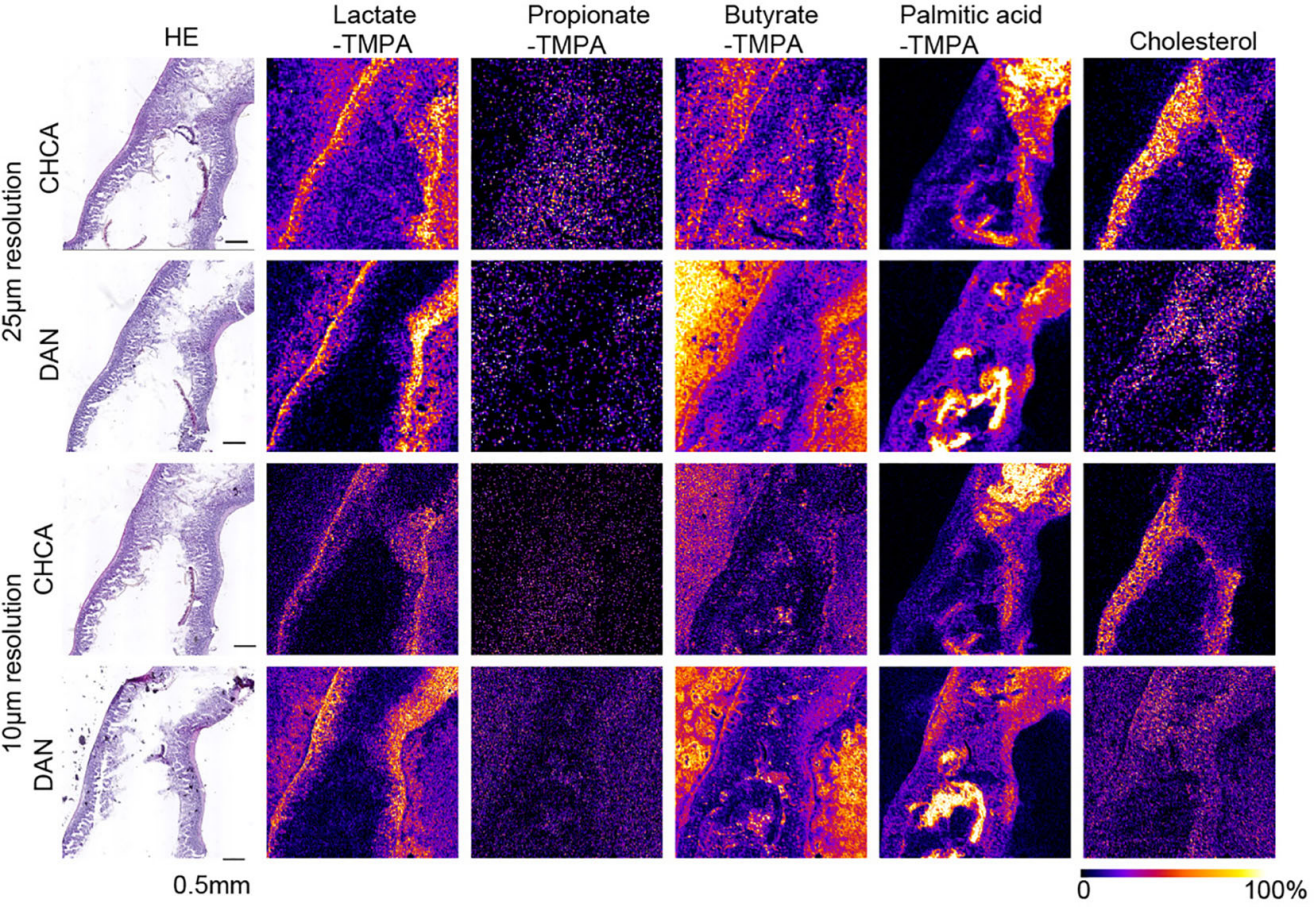
MS images at 25 µm and 10 µm resolution of TMPA-conjugated lactate, Propionate, Butyrate, palmitic acid, and [M-OH]^+^ cholesterol in the mouse small intestine, coated with alpha-CHCA and DAN, along with H&E- stained photographs. The scale bar represents 0.5 mm. All MS images were generated with theoretical *m/z* +/- 0.05 u.

In previous studies, to avoid the formation of condensation products between carboxyl-containing matrices and TMPA, carboxyl-containing matrices such as DHB or CHCA were not used for the detection of carboxyl-amine condensation products in MALDI-MSI after on-tissue reactions (Sun et al., 2020). Instead, matrices without carboxyl groups, such as 1,5-DAN (Sun et al., 2020), norharmane (Kaya et al., 2023), and 9-aminoacridine (Krivosheina et al., 2021), were used. However, these matrices contain amino groups that can generate significant matrix-derived interference peaks during positive ion mode measurements. In our experiments, after performing the on-tissue derivatization reaction with TMPA, a large interference peak was detected at a position slightly shifted from the theoretical *m/z* value of butyrate-TMPA (*m/z* 242.223) to *m/z* 242.231 in mouse cecal tissue sections sprayed with 1,5-DAN. This interference peak hindered accurate determination of the butyrate-TMPA distribution (**Figure 2**, **Supplementary Figures 2**, **3**). To apply carboxyl-containing matrices commonly used for positive ion detection in MALDI-MSI, both the matrix and endogenous carboxylic acids were derivatized into quaternary amines using TMPA and HATU under AcCN gas. After complete evaporation of the organic solvent from the OTCD-treated tissue section, CHCA was applied by sublimation. Upon examination of the MALDI-MSI spectra, CHCA-derived interference ions strongly detected at *m/z* 335.1026 ([2M-CO_2_+H]^+^), but interference ions corresponding to the theoretical *m/z* value of the CHCA-TMPA condensation product (*m/z* 343.213) were minimal and did not interfere with the detection of endogenous carboxylic acids (**Supplementary Figure 4**).

**Figure 3:**
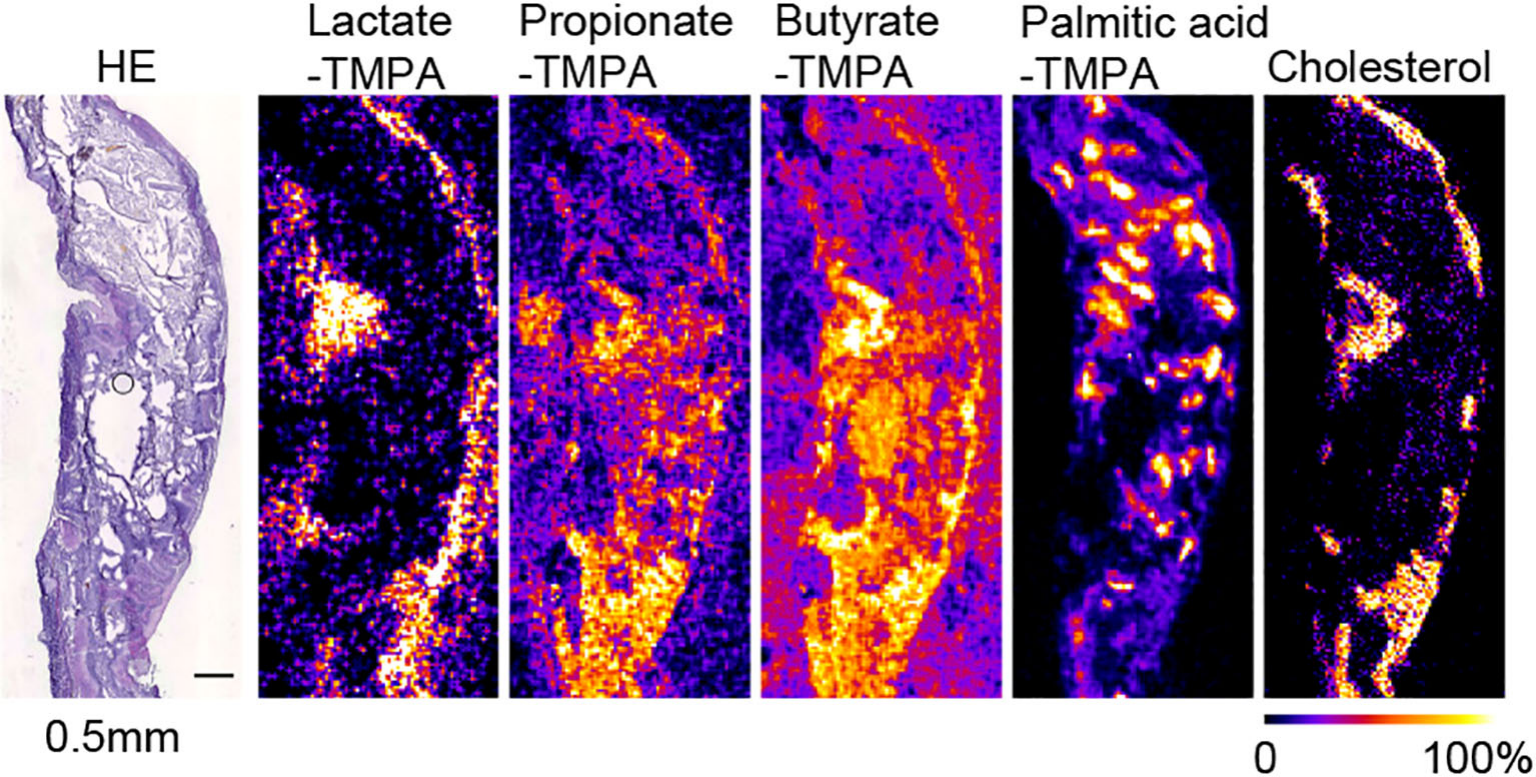
MS images at 50 µm resolution of TMPA-conjugated lactate, propionate, butyrate, palmitic acid, and [M-OH]^+^ cholesterol in the mouse cecum, coated with alpha-CHCA, along with H&E-stained photographs. The scale bar represents 0.5 mm. All MS images were generated with theoretical *m/z* +/- 0.05 u.

In mouse cecal tissue, the distribution of lactate-TMPA corresponded to the smooth muscle that forms the intestinal wall. Smooth muscle is known to obtain energy through glycolysis, and lactate is a metabolite produced as a result of glycolysis (**Figure 3**). Similarly, the TMPA derivative of lactate was detected in the small intestine. To confirm its exact distribution, the laser pitch was adjusted, and analyses were performed at spatial resolutions of 10 and 25 microns (**Figure 2**). The results showed strong detection of lactate in the smooth muscle and weak detection in the villi (**Figure 2**). TMPA derivatives of propionic acid and butyric acid, which are microbial metabolites, were strongly detected in the cecum (**Figure 3**). When comparing 50 μm resolution mass images of the cecum with HE-stained tissue images, darkly stained areas were observed between epithelial cells and undigested food residues, and strong signals derived from propionic acid and butyric acid were detected in those areas (**Figure 3**). On the other hand, signals derived from SCFAs were minimal in the small intestine (**Figure 2**). Propionic acid and butyric acid are representative SCFAs produced by intestinal bacteria, and this is consistent with the fact that the anaerobic bacteria that produce them are abundant in the cecum but almost absent in the small intestine.

The distribution of these metabolites corresponded to the dark blue staining of intestinal contents (bacteria) with hematoxylin after HE staining in the raster scan (**Figures 2**, **3**). On the other hand, dietary long-chain fatty acids showed strong signals in regions corresponding to food residues distribution in the intestine. When CHCA was used as the matrix and analysis was performed in the positive mode, a signal was observed for the dehydrated ion of cholesterol (*m/z* 369.351). Since cholesterol does not react with TMPA in carboxylic acid-amine coupling reactions. In the cecum, cholesterol was localized to intestinal epithelial cells, while in the small intestine it corresponded to that of villous epithelial cells (**Figures 2**, **3**).

### Identification of intestinal bacteria

We collected fresh intestinal contents from the cecum of mice of the same strain (C57BL/6N) that were raised under the same environment as the mice used in the MALDI-MSI experiments and performed anaerobic culture using Brucella HK agar medium. We estimated the number of bacteria by performing serial dilution using the Miles and Misra method (Slack and Wheldon, 1978). Mass spectra were obtained for three kinds of colonies of different shapes that were picked up and spread on the target plate. We identified bacterial species by comparing peak information obtained from MALDI-MS analyses with theoretical molecular weights derived from prokaryotic nucleic acid sequences using MicrobialTrack software (Shimadzu Corp., Kyoto, Japan) (Sekiguchi et al., 2023). The identified bacterial species were, in order of abundance, *Bacteroides acidifaciens Ligilactobacillus murinus*, and *Lactococcus* sp*002492185*. (**Table 3**) Assigned ribosomal subunit proteins of isolated colonies were listed in **Tables 4**–**6**.

**Table 3:** The types of strains identified using MALDI-TOF MS from colonies cultured anaerobically from the contents of the murine cecum, identification scores, and bacterial counts are shown.

Identification	Confidence score	Log CFU/mL
*Bacteroides acidifaciens*	15<	8.00
*Lactococcus* sp*002492185*	15<	6.48
*Ligilactobacillus murinus*	15<	7.85

**Table 4:** A list of observed ribosomal proteins from *Bacteroides acidifaciens*.

*m/z* detected	Peak intensity	Predicted protein name	[M+H]^+^(avg.)	Amino acid sequence
4,588.6	1.475	50S ribosomal protein L36	4,587.5	MKVRASLKKRTPECKIVRRNGRLYVINKKNPKYKQRQG*
6,290.4	1.399	50S ribosomal protein L30	6,288.5	MSTIKIKQVKSRIGAPADQKRTLDALGLRKLNRVVEHESTPSILGMVDKVKHLVAIVK*
6,305.3	1.172	50S ribosomal protein L34	6,304.3	MKRTFQPSNRKRKNKHGFRERMASANGRRVLAARRAKGRKKLTVSDEYNGQKW*
7,291.5	0.429	50S ribosomal protein L35	7,289.7	MPKMKTNSGSKKRFTLTGTGKIKRKHAFHSHILTKKSKKRKRNLCYSTTVDATNVSQVKELLAMK*
7,629.0	1.312	50S ribosomal protein L29	7,626.8	MKIAEIKEMTTSDLVERVEAETANYDQMVINHSISPLENPAQIKQLRRTIARMKTELRQRELNNK*
9,173.7	0.266	50S ribosomal protein L27	9,172.5	MAHKKGVGSSKNGRESQSKRLGVKIFGGEACKAGNIIVRQRGTEFHPGENM GMGKDHTLFALVDGTVNFKVGKGDRRYVSVVPATEA*
9,807.0	1.467	30S ribosomal protein S19	9,806.5	MSRSLKKGPYINVKLEKRILAMNESGKKVVVKTWARASMISPDFVGHTVAVHNG NKFIPVYVTENMVGHKLGEFAPTRTFRGHAGNKKK*
9,960.3	0.306	30S ribosomal protein S17	9,959.7	MEARNLRKERTGVVLSNKMDKTITVAAKFKEKHPIYGKFVSKTKKYHAHDEKNECN IGDTVSIMETRPLSKTKRWRLVEIIERAK*
10,546.5	0.150	30S ribosomal protein S18	10,545.6	MAQQVQSEIRYLTPPSVDVKKKKYCRFKKSGIKYIDYKDPEFLKKFLNEQGKILPRRITGT SLKFQRRIAQAVKRARHLALLPYVTDMMK*
11,290.1	0.314	30S ribosomal protein S10	11,288.2	MSQKIRIKLKSYDHNLVDKSAEKIVRTVKATGAIVSGPIPLPTHKRIFTVNRSTFVNKKSR EQFELSSYKRLIDIYSSTAKTVDALMKLELPSGVEVEIKV*
11,700.1	0.419	50S ribosomal protein L24	11,698.6	MSKLHIKKGDTVYVNAGEDKGKTGRVLKVLVKEGRAFVEGINMVSKSTKPNAKNP QGGIVKQEASIHISNLNPVDPKTGKATRIGRKVSSLEGKRTVVRYSKKSGEEIK*
12,536.5	0.298	50S ribosomal protein L7/L12	12,535.3	MADLKAFAEQLVNLTVKEVNELATILKDEYGIEPAAAAVAVAAGPAAGAAAVEEKTSFDV VLKSAGSAKLQVVKAVKEACGLGLKEAKDLVDGAPSTVKEGLAKDEAESLKKTLEEAGAEVELK*

**Table 5:** A list of observed ribosomal proteins from *Lactococcus* sp*002492185*.

m/z detected	Peak intensity	predicted protein name	[M+H]^+^(avg.)	Amino acid sequence
6,007.4	0.481	50S ribosomal protein L33	6,008.1	MRVNITLEHKESGERLYLTQKNKRNNPDRLELKKYSPKLRKHVIFKEVK*
6,207.7	8.853	50S ribosomal protein L30	6,208.4	MAQIKITLVKSPIGRIPAQRKTVVALGLGKLGSSVVKEDNAALRGMANSISHLVTIEEVK*
6,567.5	5.899	50S ribosomal protein L32	6,568.5	MAVPARRTSKSKKNKRRTHYKMTAPTVTFDETTGDYRHSHRVSLKGYYKGRQVRDAK*
7,069.9	5.711	50S ribosomal protein L28	7,070.2	MSKECYFTGRKTVSSNNRSHAMNQTKRVVKPNLQKVQILENGELKTVWASAK ALKKLPAGVERV*
7,943.2	4.430	50S ribosomal protein L29	7,943.3	MKLNETKSLLKDLRALSIDELATREAELKKELFELRFQAAAGRLENTAKLDEVKKTIARV KTVQRELTK*
8,128.9	4.894	30S ribosomal protein S20	8,128.3	MANIKSAIKRAELNVAANERNSQQKSAMRTAIKKFEKAPSEDTYKAASSAIDKAASKGL IHANKASRDKSRLAAKLG*
9,179.2	3.691	30S ribosomal protein S18	9,177.8	MAFQKRGGFKRRKKVDFIAANKIEVVDYKDTELLKRFISERGKILPRRVTGTSAKNQ RKVVTAIKRARVMALLPFVASDEN*
10,178.3	0.605	30S ribosomal protein S17	10,177.8	MERNQRKVYQGRVVSDKMDKTITVVVETKRNHPVYGKRINYSKKYKAHDENNTA KTGDIVRIMETRPLSKDKRFRLIEIVEEAVII*
10,302.7	1.655	30S ribosomal protein S16	10,301.9	MSVKIRLTRMGSKKKPYYRINVADSRSPRDGRFIETVGTYNPLVAENQVTLKEERV MEWLNNGAQPSDTVRNILSKAGIMKKFHEQKYSK*
10,348.2	1.448	30S ribosomal protein S15	10,347.0	MAISKEKKQEIIKQYARTEGDTGSPEVQIAVLTWEINHLNDHIKAHKKDHATYRG LMKKIGHRRNLLAYLRKKDVQRYRELIASLGLRR*
10,460.6	1.568	30S ribosomal protein S19	10,460.0	MSRSLKKGPFADEHLMKKVEAQENAEKKSVIKTWSRRSTIYPNFVGLTIAVYDGRK HVPVYVQEDMVGHKLGEFAPTRTYRGHAADDKKTRR*
10,801.2	1.329	50S ribosomal protein L24	10,799.7	MFVKTGDTVKVIAGKDRGTTGKVIKALPKVNKVIVEGVAIVKKHQKPDAVNPNGA ILEIEAPIHVSNVQVLDKNGVAGRVGYKEVDGKKVRFNKKSGEVLD*
10,910.4	0.539	50S ribosomal protein L23	10,909.6	MSLYDVIRKPIITEASMQAMDQKKYTFEVDARAHKLLIKQAVEAAFEGVQVASVNT ISVKPKAKRVGRYTGFKPGYKKAIVTLTEGSKSIDLFGEGEDAE*
11,613.1	0.603	30S ribosomal protein S10	11,611.5	MATKKIRIRLKAYEHRILDAAAEKIVETAKRTNAEVSGPIPLPTDRSVYTVIRATHKYK DSREQFEMRTHKRLIDIIEPTQKTVDSLMKLDLPSGVNIEIKL*
12,304.4	0.340	50S ribosomal protein L22	12,303.3	MAEITSAKATARTVRVSPRKTRLATDLIRGKRVADAIAILKFTPTKGAAEVLKV MNSAIANAENNFGLEKANLVVSETFVNEGPTMKRFRPRAKGSASPINKRTSHITVVVAEKE*
12,457.1	0.524	50S ribosomal protein L18	12,454.3	MISKPDKNKLRQKRHIRVRGKISGTAETPRLNVFRSNTNIYAQVIDDEAAVTL ASASSLKLTGTKTEQAAEVGKLIAEAAKAKGIEAVVFDRGGYLYHGRVQALAEAAREAGLKF*
13,060.4	0.176	50S ribosomal protein L19	13,060.2	MSISLIDSINAGQLRSDIPEFRPGDTVRVHAKVVEGTRERIQMFEGVVIARKGS GISETYTVRKISNGVGVERIFPLHTPRVEKIEVIRHGRVRRAKLYYLRALQGKAARIPERRK*
13,174.6	0.212	30S ribosomal protein S11	13,173.1	MAKITRKRRVKKNIETGIAHIQSTFNNTIIMITDVHGNALAWSSAGSLGFKGSKKSTPFAAQM ASEAAAKAAQEQGLKTVSVTVKGPGSGRESAIRALAAAGLNVTSISDVTPVPHNGARPPKRRRV*
13,358.6	0.395	30S ribosomal protein S13	13,358.4	MARFAGVDIPNEKRIVISLTYVYGVGLQTAKKVLAAAGVSEDVRTKDLTSDQEDAIRRELDG LKLEGDLRREVNLNIKRLMEIGSYRGMRHRRGLPTRGQNTKNNARTRKGPAKAIAGKKK*
13,973.5	0.470	30S ribosomal protein S9	13,971.1	MAQVQYAGTGRRKNSVARVRLVPGTGKIVINKRDVEDYIPQAALRLVINQPFA ATQTEGSYDTLVNVNGGGVSGQSGAIRHGIARALLEVDPDFRGALK RAGLLTRDARMVERKKAGLKKARKASQFSKR*
15,000.9	0.471	30S ribosomal protein S12	15,002.5	MPTINQLVRKGRHSKVEKSNSPALNIGYNSRKKLQTKVASPQKRGVATRVGTM TPKKPNSALRKFARVRLSNLIEVTAYIPGIGHNLQEHSVVLLRGGR VKDLPGVRYHIVRGALDTAGVTDRKQSRSKYGTKKPKA*
15,718.9	0.186	50S ribosomal protein L15	15,718.0	MKLHEMKAAEGSRKVRNRVGRGTSSGNGKTSGRGQKGQKSRSGGGVRPGFEGGQTEL FRRLPKRGFTNVNRKEYAIVNLDTLNRLGDGAEVTAETLVAAKIIKDVKSGIKVLANGE LTVKNLKVNVAKASAAAKAAIEAAGGSVETLEAK*

**Table 6:** A list of observed ribosomal proteins from *Ligilactobacillus murinus*.

*m/z* detected	Peak intensity	predicted protein name	[M+H]^+^(avg.)	Amino acid sequence
5,391.9	0.873	50S ribosomal protein L34	5,391.4	MKRTYQPKKRHRQRVHGFRKRMSTSNGRNVLARRRRKGRKVLSA*
6,375.1	2.304	50S ribosomal protein L30	6,372.5	MANLKVTLVRSVIGRPQNQREIVKGLGLGRVNSSVVVPDNAA MRGAIRKINHLVDVELAK*
6,820.4	1.727	50S ribosomal protein L28	6,819.0	MAKDFVTGRKTTFGKKRSHALNQTNRSWKPNLQKVRILVDGKPKKVWVSARALKSGKVTRV*
7,590.7	0.237	50S ribosomal protein L35	7,588.0	MPKQKTHRASAKRFKRTGNGGLKRAHAFTSHRFHGKTKKQRR QLRKPAMVSASDMKRIKQMLSQMK*
7,622.5	1.606	50S ribosomal protein L29	7,620.9	MKINEINELTTAEMLEKEKQFKEELFNLRFQLATGQLENTARLKEVRKTIARIKTALRQQELNK*
7,921.5	1.334	50S ribosomal protein L24	7,918.3	MFIKSNDKVKVIAGKDKGKEGVVIKAFPANDRVIVKGVNIVKKHQKPNN ANPNGGIVEMEAPIHVSNVKKISE*
9,009.2	0.747	30S ribosomal protein S18	9,007.5	MPQQRRGGRRRRKVDFIAANHIEYIDYKDTDLLKRFISERGKILPRRV TGTSAKNQRKLTVAIKRARIMGLLPFVAED*
9,140.8	0.521	30S ribosomal protein S20	9,138.6	MPIIKSAIERVKTNEKANARNSAELSKMRTAIKKFEKAKTAGAEDVEKL YREAVSAVDRAHSKGLIKANKAARDKSRMAARLAK*
9,476.4	0.237	50S ribosomal protein L31 type B	9,475.5	MKQGIHPDYHKVVFMDSATGYKFLSGSTKTTEETIEWEDGNTYPLIRVEISS DSHPFYTGKQKFTQADGRVDRFNKKYGFTN*
10,120.0	0.365	30S ribosomal protein S17	10,117.8	MSEGRNQRKVYQGRVVSDKMEKTITVVVETYVNDKVYGKRVKYSKKY KAHDENNEAKVGDIVKIMETRPLSATKRFRLLEIVEKAVII*
10,259.8	0.493	30S ribosomal protein S15	10,257.8	MAISNEKKNEIMKKYARHEGDTGSAEVQIAVLTADINELNEHVRSHK KDFASQRGLMKKIGHRRNLLAYLRNKDVQRYRELIKSLGLRR*
10,343.0	0.373	30S ribosomal protein S16	10,340.0	MSVKIRLKRMGSKKRPFYRIVVADSRSPRDGRFIETVGTYNPLTQPEQ VTLKEEAIMGWLNNGAQPSDTVRNILSKEGVMKKFHEAKYSKK*
10,518.6	0.339	30S ribosomal protein S19	10,517.1	MSRSLKKGPFVDEHLMKKVEAQADQEKKSVIKTWSRRSTIFPSFIGYTIAVYDGR KHVPVYIQEDMVGHKLGEFVPTRTFHGHAADDKKTGKK*
11,228.3	0.274	50S ribosomal protein L23	11,226.0	MESRDVILRPIITEASMNEMDNKRYTFEVDLRANKTQVKDAVEDIFEVEVAKVN IMNVKGKKKRMGRYEGYTRKRRKAIVTLKPESKDIQLFNEE*
11,653.0	0.214	30S ribosomal protein S10	11,648.6	MAKQKIRIRLKAYEHRILDQSADKIVETAKRTGAQISGPIPLPTERTIYTVIRSPH KYKDSREQFEMRTHKRLIDIVNPTPKTVDSLMKLDLPSGVDIEIKL*
12,242.8	0.221	50S ribosomal protein L7/L12	12,240.0	MALDTEKIIADLKEASILELNDLVKAIEEEFGVSAAAPVAAAGAAAGAAEEKS EFDVELTDPGAGKVKVIKAVKDITGLGLKDAKGLVDGAPSVIKEGVAKEEA EEIQAKLEEVGAKVTLK*

**Figure 4:**
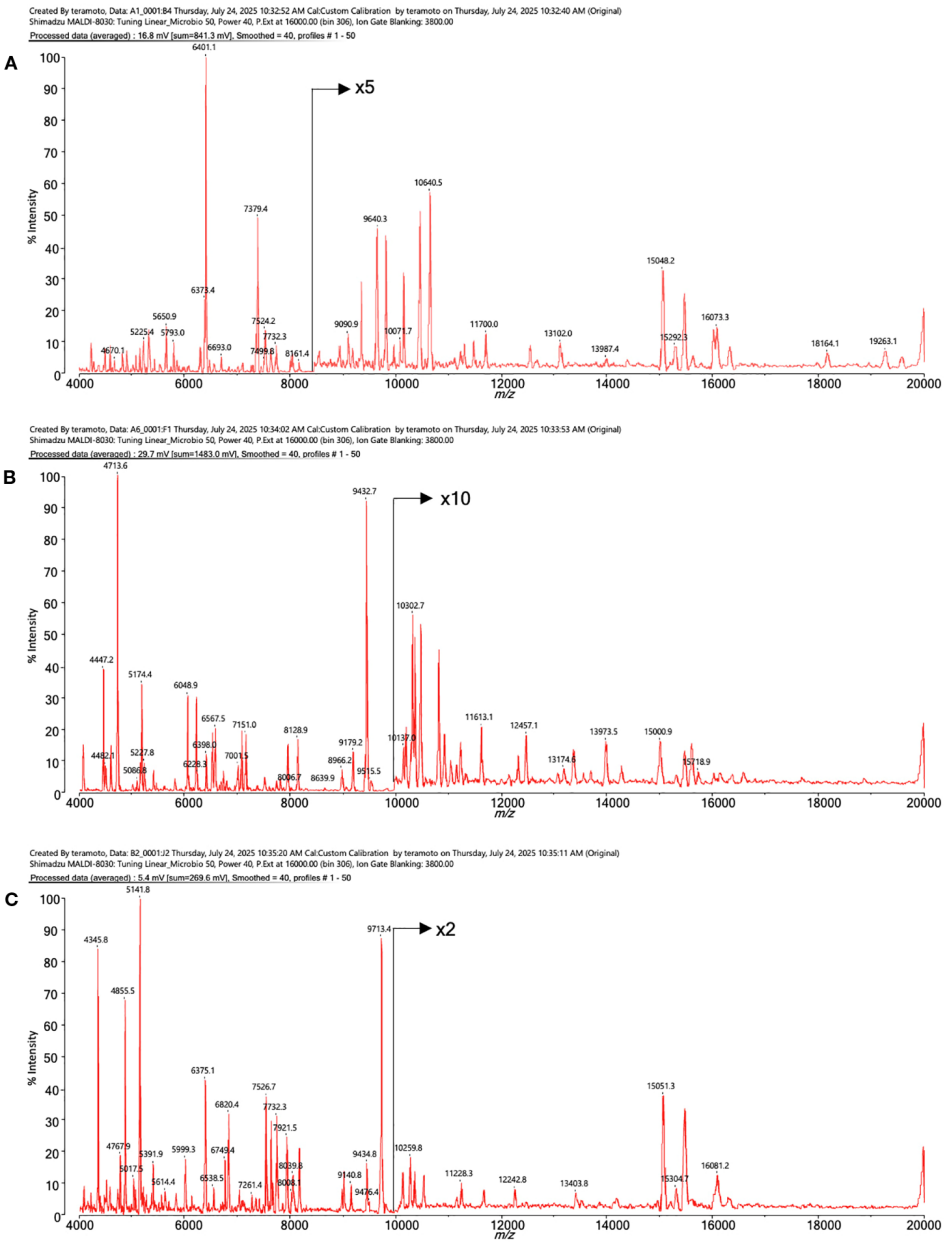
The MALDI mass spectra of proteins from intestinal bacteria. **(A)** *Bacteroides acidifaciens*, **(B)** *Lactococcus* sp002492185, **(C)** *Ligilactobacillus murinus*.

**Figure 4** shows the MALDI mass spectra of identified three different shaped colonies.

The *Bacteroides* genus is one of the most important bacterial genera in the human and mouse intestines and is known to break down dietary fiber and produce short-chain fatty acids (Markowiak-Kopec and Slizewska, 2020). The remaining two species were identified as *Lactococcus* sp*002492185* and *Ligilactobacillus murinus*, both of which are lactic acid bacteria. These bacteria are known to metabolize dietary fiber and produce SCFAs (**Table 3**). The spectra data were submitted to jPOST repository (Okuda et al., 2025) under accession number JPST004050 (PXID: PXD067872) https://repository.jpostdb.org/entry/JPST004050, accessed on 29 August 2025).

## Discussion

Among the organic acids present in the gastrointestinal tract, SCFAs produced by intestinal bacteria are volatile (Myhrstad et al., 2020). While the volatilization of butyrate and propionate is minimal in frozen samples, losses occur during air drying at room temperature after frozen sectioning. Therefore, we immediately thaw-mounted frozen samples onto ITO-coated slides after cryosectioning and performed freeze-drying at -20°C, avoiding evaporation. By chemically derivatizing the volatile carboxylic acid metabolites *in situ* and converting them into quaternary amine-bound compounds, detection by MALDI-MSI became possible. This sample preparation method differs from those used in previous studies, where volatile carboxylic acids were undetectable (Sun et al., 2020), likely explaining their inability to detect volatile SCFAs.

Next, molecules containing two or more carboxyl groups were investigated. In liquid-phase reactions involving polycarboxylic acids, TMPA, and the condensing agent HATU, LC-MS analysis confirmed the coexistence of molecules with all carboxyl groups derivatized, some with two derivatized groups, and others with only one derivatized carboxyl group. However, when the reaction products were analyzed as droplets by MALDI-MS, only monovalent ions with a single derivatized carboxyl group were detected. This result indicates that MALDI-MS is not suitable for the detecting molecules with two quaternary amines attached. This observation is consistent with MALDI-MSI experiments on tissue sections, where polycarboxylic acid molecules were detected as having only one derivatized carboxyl group (Sun et al., 2020).

Subsequently, we investigated the matrix used for MALDI-MSI analysis. Previous studies used 1,5-DAN, which contains amino groups, as the matrix after performing a condensation reaction between carboxyl-containing compounds in tissue sections and the amine group of TMPA (Sun et al., 2020). Using the same method, we observed a contamination peak, likely a reaction by-product, present at an *m/z* value close to the theoretical *m/z* value of TMPA-butyrate. Although the distribution of TMPA-butyrate has not been reported in previous studies, we were also unable to elucidate the distribution of TMPA-butyrate using this method (Sun et al., 2020). Therefore, we employed an alternative matrix. Compounds such as 2,5-DHB or CHCA, which contain carboxyl groups, are suitable for detecting endogenous quaternary amines. However, when matrix solutions were applied after on-tissue derivatization, residual TMPA and HATU reacted with the matrix compounds, generating significant amounts of condensation products. To address this issue, we changed the matrix application method to solvent-free sublimation and attempted to detect SCFAs derivatized with TMPA. This approach eliminated contamination peaks, and the distributions of butyrate-TMPA and propionate-TMPA were successfully elucidated.

In this study, we were unable to quantify SCFA present in frozen tissue. Due to difficulties in handling volatile substances contained in tissue sections, the conventional method of uniformly applying the same substance labeled with stable isotopes and comparing the signal intensity obtained from endogenous substances cannot be used to ensure quantitative accuracy in MALDI-MSI. This is because spraying the solvent onto the sample causes the volatile organic acids contained in the frozen section to evaporate. To determine the concentration of volatile organic acids in intestinal contents, it is best to extract the intestinal contents from the remaining frozen block after MALDI-MSI analysis and analyze them using methods that can accurately quantify them, such as GC-MS or LC-MS.

Following MALDI-MSI analysis, HE staining of the slides revealed that the distribution of short-chain fatty acids (SCFAs) in the cecum corresponded with hematoxylin-positive areas. These areas are likely associated with intestinal bacteria. Conversely, no SCFA-TMPA signals other than lactic acid were detected in the small intestine; only undigested residues and small intestinal tissue were present hematoxylin-positive. The small intestine contains very few bacteria compared to the lower digestive tract. In healthy organisms, anaerobic bacteria that produce SCFAs are almost absent from the small intestine (Khoshini et al., 2008). In this study, intestinal bacteria were collected from the cecum of mice, identified, and quantified using a stepwise dilution method and anaerobic culture. All of the detected and identified bacteria were species involved in short-chain fatty acid production, and these results were consistent with those obtained by MALDI-MSI detection of short-chain fatty acids. However, it was not possible to obtain detailed information on the distribution of the bacteria. Future studies using fluorescence *in situ* hybridization (FISH) analysis with FISH probes targeting the identified bacteria on sections after MALDI-MSI will allow for a more detailed analysis of the distribution of intestinal bacteria and metabolites.

## Data Availability

The datasets presented in this study can be found in online repositories. The names of the repository/repositories and accession number(s) can be found in the article/**Supplementary Material**.

## Supplementary material

Supplementary Figure 1 Representative MS/MS spectrum of TMPA-derivatized citric acid. Red arrows indicate characteristic *m/z* values corresponding to the number of TMPA-derivatized carboxyl groups.

Supplementary Figure 2 Mass spectrum near the theoretical *m/z* value of butyrate-TMPA. A foreign peak very close to the theoretical value of butyrate-TMPA was detected when derivatization was performed using a hand spray method and 1,5-DAN solution was applied by airbrush as the matrix. In contrast, when CHCA was applied by sublimation after chemical derivatization, no foreign peak was observed, and a peak of butyrate-TMPA at the theoretical value was observed.

Supplementary Figure 3 MS images at 50 µm resolution of TMPA-conjugated butyrate in the mouse cecum, coated with 1,5-DAN solution, along with and H&E-stained photographs. The scale bar represents 0.75 mm. The MS image was generated with theoretical *m/z* +/- 0.05 u.

Supplementary Figure 4 Averaged mass spectrum between *m/z* 200 to 500 from TMPA-treated and CHCA sublimated mouse colon tissue section. No peak was found at theoretical CHCA-TMPA ligated molecular weight at *m/z* 343.213.
