# Supplementary material for: On-tissue derivatization for mass spectrometry imaging reveals the distribution of short chain fatty acids in murine digestive tract
Source: Front Cell Infect Microbiol. 2025 Oct 3;15:1584487. doi: 10.3389/fcimb.2025.1584487 (PMC12531170; doi:10.3389/fcimb.2025.1584487)

Supfig2

Theoretical butyrate-TMPA *m/z* 242.2225

TMPA-hand spray
DAN-hand spray

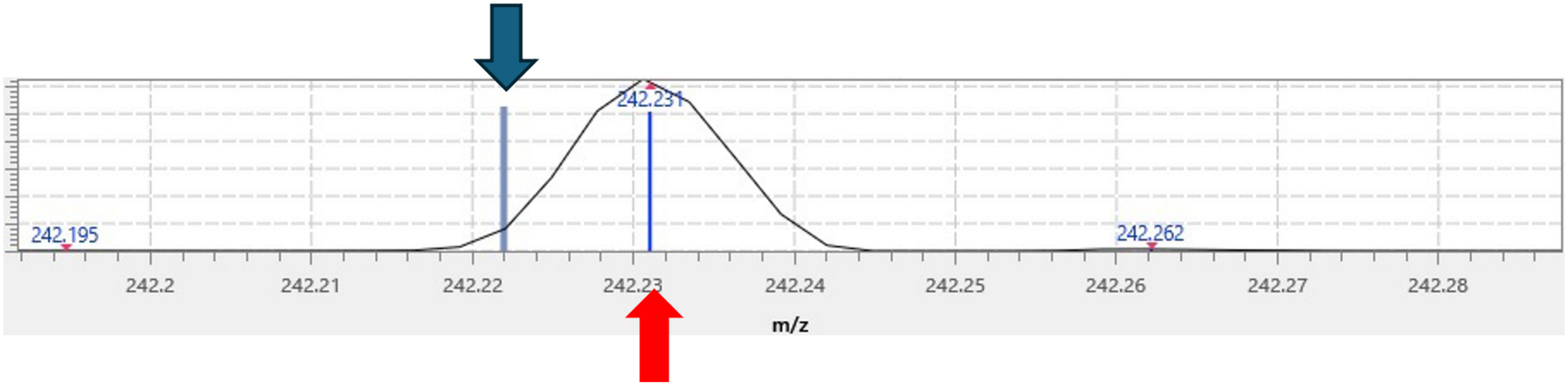

Contaminant peak

Theoretical butyrate-TMPA *m/z* 242.2225

TMPA-pneumatic sprayer
CHCA-Sublimation

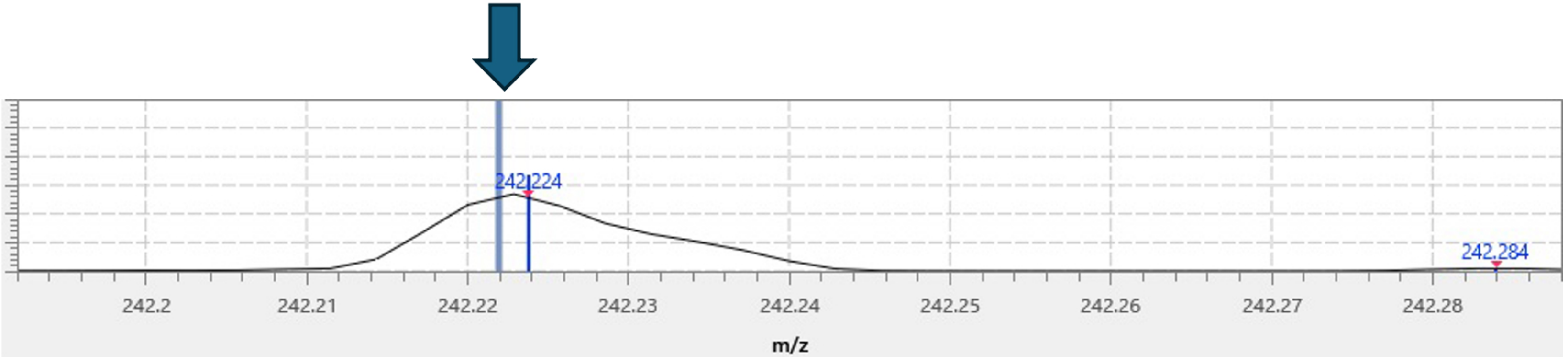

Supplement: Supplementary Figure 1 — Representative MS/MS spectrum of TMPA-derivatized citric acid. Red arrows indicate characteristic m/z values corresponding to the number of TMPA-derivatized carboxyl groups. [file Image1.pdf]
