# Supplementary figures and images for: On-tissue derivatization for mass spectrometry imaging reveals the distribution of short chain fatty acids in murine digestive tract
Source: Front Cell Infect Microbiol. 2025 Oct 3;15:1584487. doi: 10.3389/fcimb.2025.1584487 (PMC12531170; doi:10.3389/fcimb.2025.1584487)

Supfig.3

HE-staining

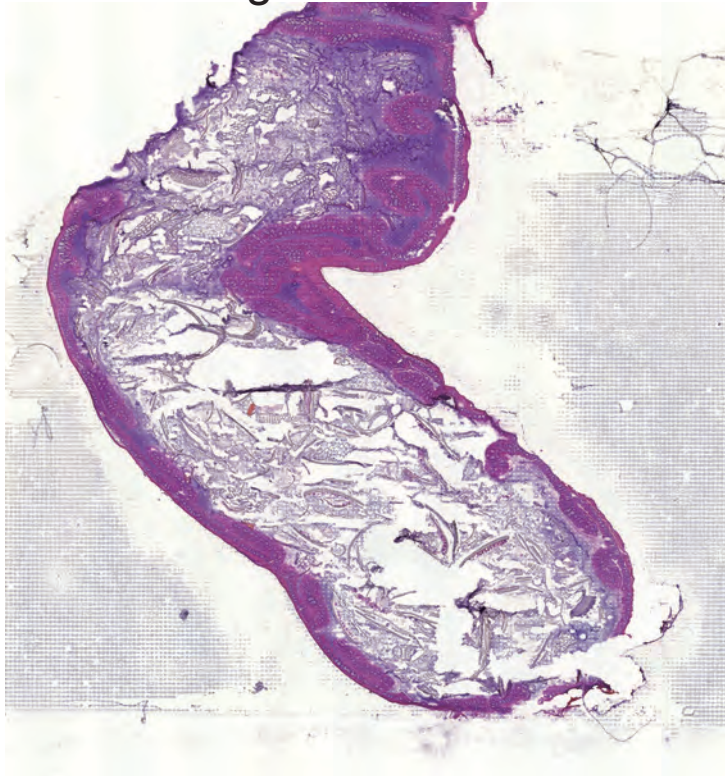

Butyrate-TMPA ( $m/z$  242.223  $\pm$  0.05)

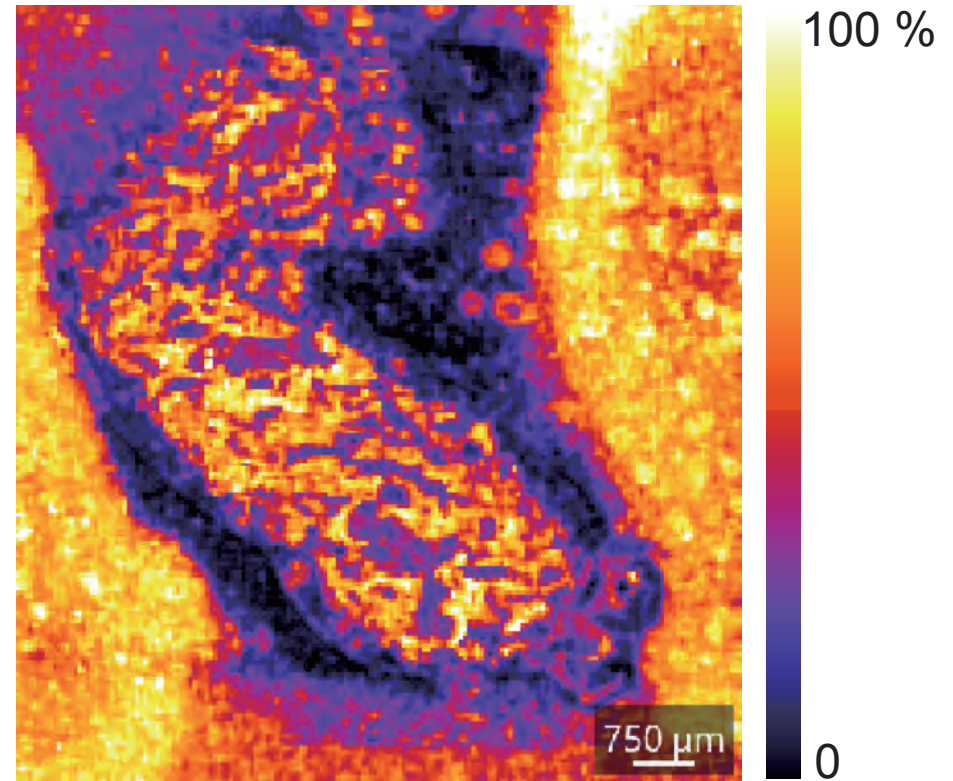

Supplement: Supplementary Figure 2 — Mass spectrum near the theoretical m/z value of butyrate-TMPA. A foreign peak very close to the theoretical value of butyrate-TMPA was detected when derivatization was performed using a hand spray method and 1,5-DAN solution was applied by airbrush as the matrix. In contrast, when CHCA was applied by sublimation after chemical derivatization, no foreign peak was observed, and a peak of butyrate-TMPA at the theoretical value was observed. [file Image2.pdf]

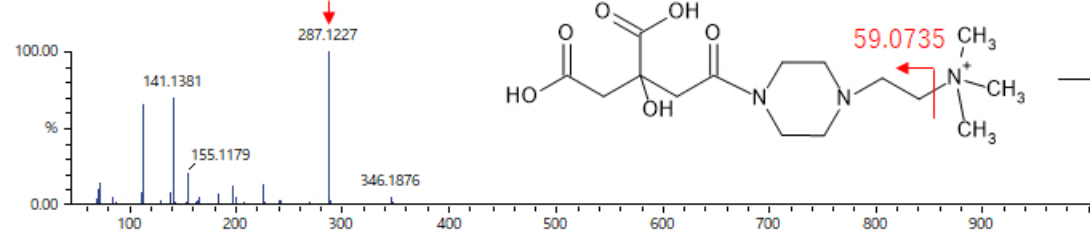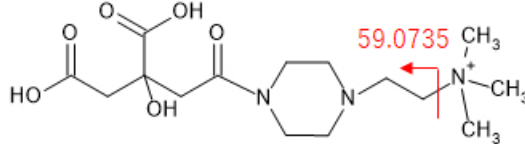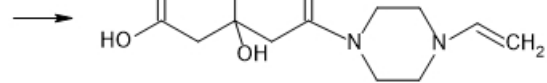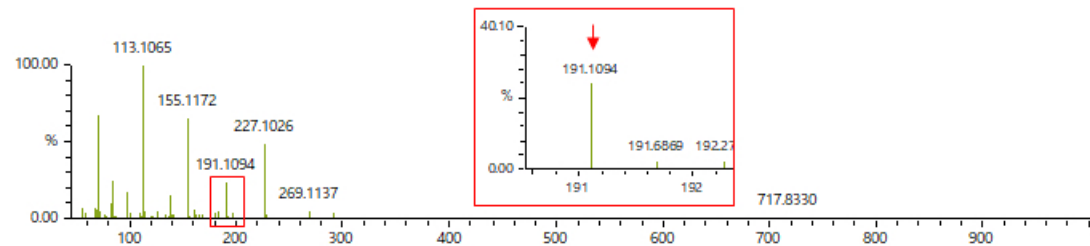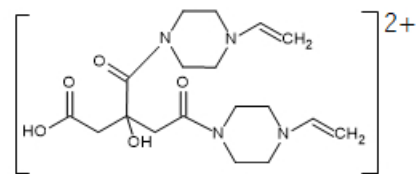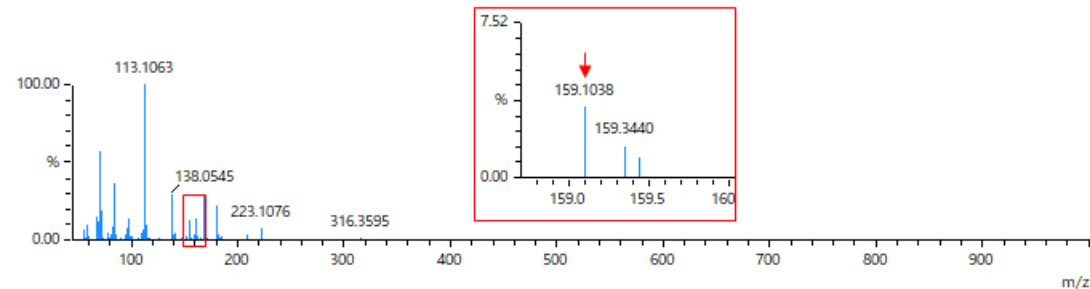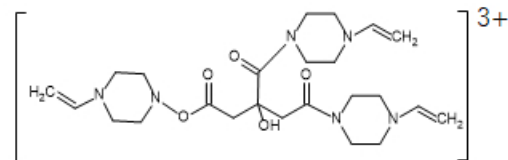

Supplement: Supplementary Figure 3 — MS images at 50 µm resolution of TMPA-conjugated butyrate in the mouse cecum, coated with 1,5-DAN solution, along with and H&E-stained photographs. The scale bar represents 0.75 mm. The MS image was generated with theoretical m/z +/- 0.05 u. [file DataSheet1.pdf]
