# Supplementary material for: On-tissue derivatization for mass spectrometry imaging reveals the distribution of short chain fatty acids in murine digestive tract
Source: Front Cell Infect Microbiol. 2025 Oct 3;15:1584487. doi: 10.3389/fcimb.2025.1584487 (PMC12531170; doi:10.3389/fcimb.2025.1584487)
Supplement: Supplementary Figure 4 — Averaged mass spectrum between m/z 200 to 500 from TMPA-treated and CHCA sublimated mouse colon tissue section. No peak was found at theoretical CHCA-TMPA ligated molecular weight at m/z 343.213. [file DataSheet2.pdf]

Supfig4

Averaged spectrum of TMPA-CHCA(sublimation) treated mouse colon section

CHCA [2M-CO_2+H]$^+$ *m/z* 335.1026

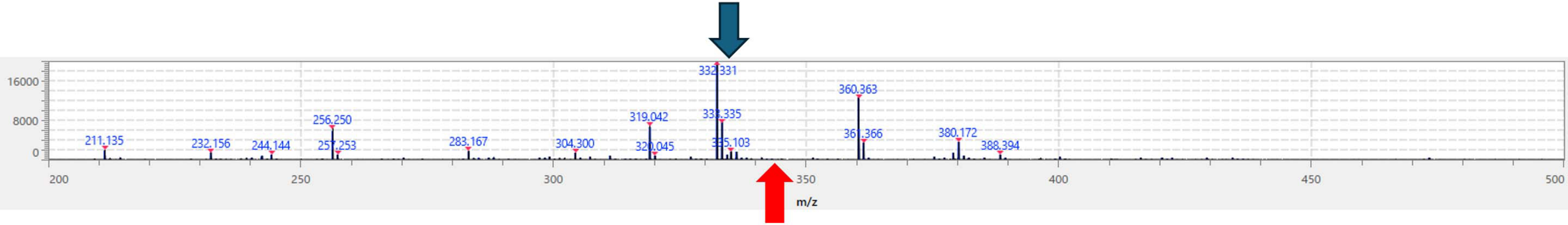

Theoretical CHCA-TMPA *m/z* 343.213
